# [*PSI*]-CIC: A Deep-Learning Pipeline for the Annotation of Sectored *Saccharomyces cerevisiae* Colonies

**DOI:** 10.1007/s11538-024-01379-w

**Published:** 2024-12-06

**Authors:** Jordan Collignon, Wesley Naeimi, Tricia R. Serio, Suzanne Sindi

**Affiliations:** 1 Department of Applied Mathematics, University of California, Merced, 5200 N Lake Drive, Merced, CA 95343 USA; 2 Department of Biochemistry and Molecular Biology, University of Massachusetts, Amherst, 240 Thatcher Rd, Amherst, MA 01003 USA; 3 Department of Chemistry and Biochemistry, University of Washington, 109 Bagley Hall, Seattle, WA 98195 USA

**Keywords:** U-Net, Image segmentation, Deep learning, Classification, Yeast, Prions

## Abstract

The $[PSI^+]$ prion phenotype in yeast manifests as a white, pink, or red color pigment. Experimental manipulations destabilize prion phenotypes, and allow colonies to exhibit $[psi^-]$ (red) sectored phenotypes within otherwise completely white colonies. Further investigation of the size and frequency of sectors that emerge as a result of experimental manipulation is capable of providing critical information on mechanisms of prion curing, but we lack a way to reliably extract this information. Images of experimental colonies exhibiting sectored phenotypes offer an abundance of data to help uncover molecular mechanisms of sectoring, yet the structure of sectored colonies is ignored in traditional biological pipelines. In this study, we present [*PSI*]-CIC, the first computational pipeline designed to identify and characterize features of sectored yeast colonies. To overcome the barrier of a lack of manually annotated data of colonies, we develop a neural network architecture that we train on synthetic images of colonies and apply to real images of $[PSI^+]$, $[psi^-]$, and sectored colonies. In hand-annotated experimental images, our pipeline correctly predicts the state of approximately 95% of colonies detected and frequency of sectors in approximately 89.5% of colonies detected. The scope of our pipeline could be extended to categorizing colonies grown under different experimental conditions, allowing for more meaningful and detailed comparisons between experiments. Our approach streamlines the analysis of sectored yeast colonies providing a rich set of quantitative metrics and provides insight into mechanisms driving the curing of prion phenotypes.

## Introduction

**Fig. 1:**
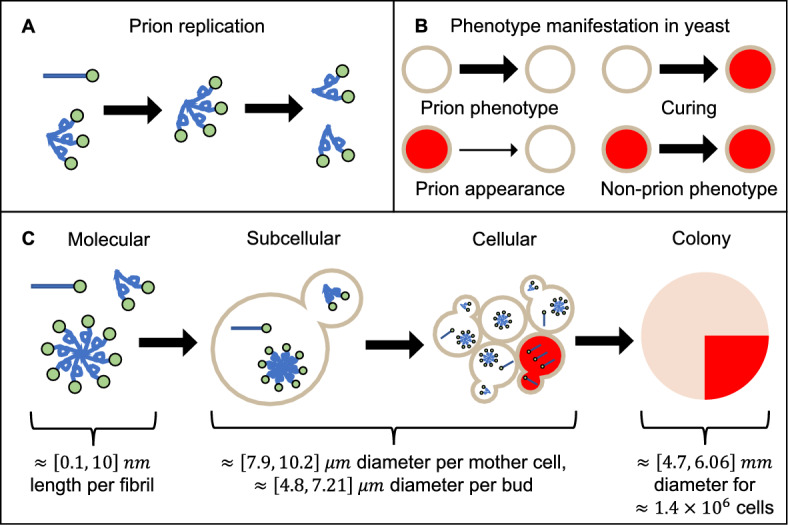
Yeast prion phenotypes are the result of multiscale processes. **a** At the molecular scale, alternatively folded proteins (twisted) act as templates that convert normally folded proteins (straight) into the alternatively folded form and assemble into aggregates. The aggregates then split into smaller segments (fragmentation) which increases the number of aggregates. **b** At the cellular scale, the presence of prion aggregates inside individual cells (represented as circles) are responsible for their white color, while the absence of prions allows pigment generation and gives them their red color. The prion phenotype could be lost sporadically, resulting in cured cells, while in rare instances-1 in $10^{6}$—(indicated by a thinner arrow) the prion phenotype appears spontaneously. **c** Phenotype expression in yeast involves multiscale processes. The dynamics inherent in protein misfolding are found at the molecular level (**a**). At the subcellular level, since prions are also found in yeast which undergo their own process of reproduction, allowing transmission of prions between attached cells. At the cellular level, the presence of prions within a cell in turn determines their phenotype (**b**). At the colony level, the collection of intercellular interactions that occur on the scale of a cell results in structured regions of one phenotype within the colony. Molecular scale was visually estimated from image data in (Kabani and Melki 2011). Subcellular and cellular scales were estimated using data from (Zakhartsev and Reuss 2018). A rough estimate for the colony scale was obtained using the minimum and maximum averaged surface area measurements of a mother cell in (Zakhartsev and Reuss 2018), multiplied by the approximate number of cells in colonies from data in (Joseph and Hall 2004) (color figure online)

Prion diseases are a class of fatal and incurable neurodegenerative diseases in mammals that include Creutzfeldt-Jacob disease, fatal familial insomnia, Gerstmann-Straussler-Scheinker syndrome, and Kuru (Prusiner 1996). Early research by Prusiner (1982, 1996) suggested that a protein-not a virus-coined as a proteinacious infectious particle-or prion-was the key infectious agent in all types of prion diseases regardless of the mammalian host, thus establishing what we know today as the prion hypothesis (Watts et al. 2006; Nowak et al. 1998). These alternatively folded proteins act as templates capable of inducing normally folded proteins of the same type to misfold (Hutti et al. 2020; Srivastava and Lapidus 2017; Hwang et al. 2009; Prusiner 1998) (see Fig. 1a). Furthermore, these alternatively folded proteins are capable of undergoing templated conversion to form aggregates (Nowak et al. 1998) which are capable of growing in size or fragmenting into smaller aggregates that induce further alternative folding, thus leading to a self-replicating aggregation process (Hutti et al. 2020; Collinge 2005). Since the formalization of the prion protein (Prusiner 1998), the study of biological processes behind prion disease and the search for appropriate solutions to eradicate them remains an active area of research.

### Yeast as a Model System

Prion proteins are not exclusive to mammals. The yeast *Saccharomyces cerevisiae* has served as a model system to understand the mechanisms underlying the appearance and progression of human diseases, including “prion-like” diseases such as Alzheimer’s (Bagriantsev and Liebman 2006; Bharadwaj et al. 2010; Smith and Snyder 2006). There are at least eight naturally occurring yeast prion proteins (Cascarina and Ross 2014; Wickner and Kelly 2016; Li and Kowal 2012) setting the stage for yeast-based platforms to help screen potential anti-prion drug candidates (Ishikawa 2021). One of the most widely studied prion protein in yeast is Sup35 which is an essential release factor in translation-termination (Tuite and Cox 2007; Lyke et al. 2019). Sup35 aggregates have the ability to self-propagate within yeast populations (Nowak et al. 1998). When grown on solid media single yeast cells grow into circular colonies containing approximately $1\times 10^{6}$ cells and exhibit a white unpigmented $[PSI^+]$ phenotype when the prion is present. In contrast, colonies that only contain the non-prion form of Sup35 exhibit the red pigmented $[psi^-]$ phenotype (Klaips et al. 2014). Spontaneous appearance of the $[PSI^+]$ phenotype is rare, occurring in approximately one in every $10^{6}$ cell divisions (Lancaster et al. 2010; Halfmann et al. 2012). Remarkably, unlike their human counterparts, the $[PSI^+]$ phenotype in yeast is reversible (Lemarre et al. 2020; Halfmann et al. 2012; Satpute-Krishnan and Serio 2005; Klaips et al. 2014). Heat shock destabilizes the prion phenotype in yeast which in time gives rise to colonies exhibiting both red and white phenotypes described as sectors (Cox 1965; Klaips et al. 2014). Figure 1 B and C summarizes the possible events determining the phenotype of newly born cells, and how the collective prion state of cells in a colony give rise to sectored phenotypes at the colony level. This type of data provides information on the prion state of a cell population and insight into changes to the prion phenotype in response to experimental treatments.

To understand how sectoring occurs in yeast, we need to consider the underlying dynamics (conversion, aggregation, and fragmentation) at the intracellular scale (Fig. 1c). Mathematical models have been proposed that explore these dynamics (Masel et al. 1999; Davis and Sindi 2016; Sindi et al. 2017) with one recently proposed to explore how multiple prion strains interact (Lemarre et al. 2019). However, such dynamics take place inside individual yeast cells that have their own biological properties (such as division which allows for transmission of proteins between attached mother-daughter cell pairs). The second phenotype occurs when a cell loses prions due to a transmission defect or destruction of existing prions within a cell (Newnam et al. 2011). As cells continue to divide over time and form a colony of thousands to millions of individual cells, phenotypic sectoring becomes observable (Klaips et al. 2014) (see Fig. 1c) indicating where subsequent daughter cells did not inherit the $[PSI^+]$ prion.

Thorough understanding of these multiscale processes may require large samples of yeast colonies under different experimental settings. This however leads to two potential problems. First, experimental settings do not always result in deterministic observable experimental output. Second, analyzing each individual colony is an extremely tedious process; colonies are often scored as sectored or pure, but there is additional information based on the size and number of sectors to help better our understanding of prion curing. For these reasons, large-scale screening involving detailed colony phenotypic analysis is unfeasible without the use of suitable instruments and algorithms capable of utilizing this information.

### The Role of Image Analysis

Technological advances have made it possible to use computational approaches to handle variation in experimental data and efficiently quantify large, complex biological datasets with the added benefit of reducing manual laboratory labor while producing outcomes comparable to manual labor. Such methods include software and image-based methods to automate microbial colony counting (Tronnolone et al. 2018; Choudhry 2016; Lamprecht et al. 2007), edge detection (Canny 1986) and for circular objects, the circle Hough transform (Hough et al. 1962; Atherton and Kerbyson 1999). With the availability of greater processing power, deep neural networks or more specifically, convolutional neural networks (CNNs) have made it possible to quickly identify objects of interest in general datasets when conventional methods are inadequate. Deep learning methods applied to images typically have one or two objectives. One class of methods seek to classify entire images by associating them with a set of user-defined classes; a couple examples of well-known models include ResNet (Tai et al. 2017) and VGG (Simonyan and Zisserman 2014; Minaee et al. 2021). Another class of methods use semantic segmentation to assign user-defined classes to each pixel in an image, rather than assigning classes to the image as a whole; such models include U-Net (Ronneberger et al. 2015) and Mask R-CNN (He et al. 2017). Methods in both cases are usually either trained from scratch or build off of a pre-trained model-such as ImageNet (Deng et al. 2009)-then re-trained on a new dataset to be applicable to specific settings. One disadvantage of using deep learning is that models often require large quantities of data. One way to address this shortcoming is through synthetic image generation to increase the size of the dataset and to address an imbalance of features in the available data. Generative adversarial networks are often used to create feature-similar images (Andreini et al. 2020) and, when images are not too complex, annotations of the image data become simpler to automate (Kruitbosch et al. 2022). It is also possible to construct computational pipelines using both classes of deep learning methods to obtain ensemble data from colony-level images of yeast. For example, the model proposed by Carl et al. (2020) segments and classifies individual yeast colonies from images of plates using both semantic segmentation and image classification and demonstrates performance superior to the tool CellProfiler (Lamprecht et al. 2007) for their scenario.

The majority of image-based models applicable to yeast however are designed for micro-colony data where individual cells are clearly visible using cell microscopy techniques, while efficient and similar models for large-scale colonies visible at eye level are less numerous. Such models however, while designed to perform specific tasks, have the implication of significantly reducing manual quantification of distinct phenotypes in laboratory settings. For example, *petiteFinder* (Nunn et al. 2023) leverages a phenotype linked to cellular respiration and long-term growth in yeast to quickly identify colonies with either phenotype present. Both traditional (Rattray et al. 2023) and hierarchical (Signoroni et al. 2023) approaches leverage contextual information for models to distinguish between different species and strains present in colony image data. Previous methods of feature detection on colony images largely utilize images where colonies appear homogeneous. One likely reason for this is that too much heterogeneity is too complex for such methods to perform well compared to homogeneous colonies. When analyzing sectored colony data however, multiple phenotypes are present experimentally and should be present when analyzing such image data. While previous methods exhibit adequate performance on counting heterogeneous colonies, they are not extensively tuned toward simultaneously quantifying and classifying heterogeneous colonies.

While deep learning methods for semantic segmentation have been developed for microscopy images of yeast such as YeastSpotter (Lu et al. 2019), YeaZ (Dietler et al. 2020), and YeastNet (Salem et al. 2021), each method is primarily optimized for cellular-level images of yeast. Carl et al. (2020) has a method grouping colonies into broad classes, but the manual annotations in the images used in this study are limited to classifying colonies into these broad classes and do not account for size and frequency of individual sectors. Sectored colony phenotypes provide a rich, chronological record of intracellular and population level prion loss events which, if quantified and mined, would provide support for uncovering the mechanisms leading to such loss events. Biologists historically have not had the tools to systematically isolate or annotate individual colonies for patterns, leaving biological data unexplored. Current biological pipelines either ignore sectored colonies or consider them all of the same type even though sectors differ by size, shape or structure of sectors observed in experiments. While advances have been made to quantify heterogeneous colony phenotypes, we do not yet have a related analysis performed on sectored *S. cerevisiae* colonies, nor do we have a dedicated toolset geared for quantifying individual sectored colonies from colony-level image data with human-comparable output. The goal of this study is to use a blend of computational tools to accelerate the ability of biologists to closely identify and explore critical subsets of colonies, starting with sectored $[PSI^+]$ *S. cerevisiae* colonies.

In this paper, we introduce [*PSI*]-CIC ([*PSI*] Colony Image Classifier) a computational pipeline to segment and quantify individual colonies of *S. cerevisiae* found in image data using both deep learning and conventional tools. In Sect. 2 we detail the [*PSI*]-CIC algorithm from segmentation of plates to classification of colonies. More specifically, we demonstrate our procedure for training a deep learning model with a U-Net architecture (Ronneberger et al. 2015) on synthetic image data, applying the trained U-Net on real colony image data, and estimating the regions of different phenotypes within each colony using information from the segmentations to obtain reasonable classifications of each colony. Sect. 3 shows results of [*PSI*]-CIC’s performance on a set of images where prion curing is induced by heat shock. Section 4 details a discussion of the [*PSI*]-CIC and how this work has an impact on the use of image segmentation in the context of prion dynamics in yeast.

## Methods

**Fig. 2:**
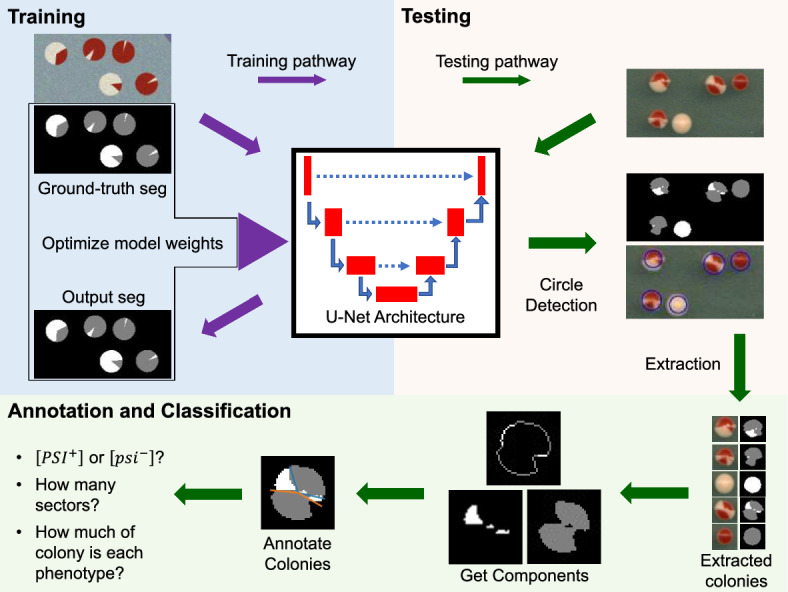
Illustration of [*PSI*]-CIC. Our proposed pipeline consists of a segmentation-classification framework, where we semantically segment images of plates containing hundreds of yeast colonies (see Sect. A.1) for the purpose of locating and classifying individual colonies. We create synthetic training images with corresponding ground-truth masks (details in Appendix A.2) used to fine-tune a modified U-Net architecture (purple arrows) (see Sect. 2.1) for performing segmentation on images of full plates. We then apply the sufficiently trained U-Net (green arrows) to segment the test images where colonies are detected (see Appendix A.3) and cropped for classification. The classification step leverages the spatial information in the segmentation to propose an annotation of the regions in the colony which is used to classify a colony as $[PSI^+]$, $[psi^-]$, or sectored (color figure online)

In Sect. 2.1, we describe the components of [*PSI*]-CIC for analyzing sectored yeast colony phenotypes (see Fig. 2). In the first component, we construct a neural network based on a U-Net architecture (Ronneberger et al. 2015) to perform image segmentation on plates containing hundreds of yeast colonies, then use the output of the network to locate and extract individual colonies. In the second component, we use image processing tools to classify colonies as $[PSI^+]$, $[psi^-]$, or sectored and estimate the frequency and shape of sectors present in each colony. Section 2.2 discusses how we train the network used in this component to recognize colonies. For this process, we detail how to incorporate synthetic training data of yeast colonies (see Appendix A.2) into the training process to both address the issue of limited annotated data available and to show its effectiveness in aiding segmentation of real colonies. Section 2.3 details how we evaluate the performance of the [*PSI*]-CIC algorithm on the annotated experimental images.

### *[PSI]*-CIC Algorithm

We follow the approach by Carl et al. (2020) and use the U-Net architecture for performing semantic segmentation on images of plates to assign a label to every pixel in the images (see Fig. 2). U-Net is a type of supervised CNN originally designed for biomedical image segmentation (Ronneberger et al. 2015; Carl et al. 2020; Overton and Tucker 2020), but is widely generalized to other segmentation tasks. For the implementation of U-Net in this paper, we modify the original architecture (Ronneberger et al. 2015) in the following way: First, we use images of size 1024$\times$1024 as input instead of size 572$\times$572. Next, we apply padding to the image before each convolutional layer to preserve the spatial resolution, which we believe is reasonable since each image almost exclusively contains background pixels on their borders. Finally, we modify the output layer such that the final segmentation is of the same spatial resolution as the input image and has three feature channels corresponding to one of three classes: background, white colony, or red colony. A softmax activation function is applied to the output of the last layer to obtain the probability of each class per pixel, then the label assigned to each pixel is the maximum probability across the three classes.

After training U-Net as described in Appendix A.4, we apply the images of interest as input to the trained U-Net and obtain segmentations of each image, then apply the resulting segmentations as input to an object detection method. Since colonies in each image appear circular by eye, we use the circle Hough transform as our method to detect colonies captured within the segmentation. Each colony detected with this method is recorded and cropped out of both the image and its segmentation for use in the classification step of [*PSI*]-CIC (See Fig. 2). Details for the implementation of the circle Hough transform is explained in Appendix A.3.

**Fig. 3:**
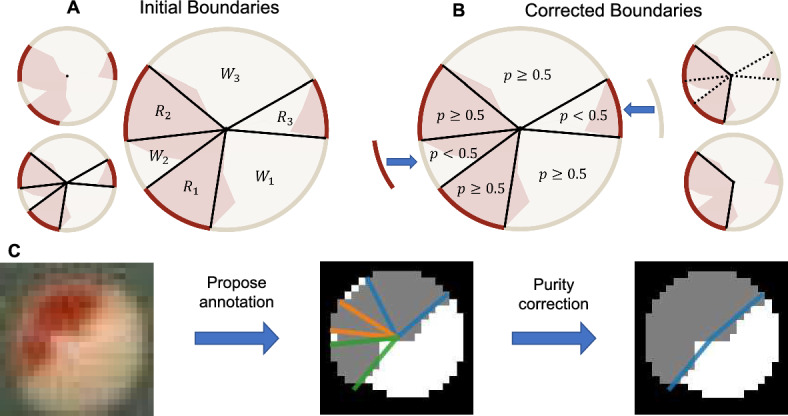
Novel annotation and sector counting procedure. Flowchart of our proposed scheme to estimate and quantify red and white regions using the process described in Sects. 2.1.1 and 2.1.2. **a** Assuming that the colony segmentation is split into red and white pixels (left top), we take the boundary of the colony and find the connected components of the red and white colony pixels respectively. We locate the endpoints of each component corresponding to the interfaces between red and white components, and for each point construct a line segment from that point to the center of the colony (left bottom). The line segments partition the entire colony into idealized regions whose color is defined by the boundary in each region (i.e. $R_1$ for red and $W_1$ for white) (right). **b** To ensure regions are consistent with their color, we use the purity metric as defined in Sect. 2.1.1 to find the proportion of pixels inside each region that have the same color as the pixels on the boundary (left). Any regions whose purity metric is less than 0.5 will have the outer boundary change color (right top). After the change, adjacent components that have the same color (regions surrounding dotted line segments) will be merged (right bottom). **c** Example of A and B applied to a segmentation of an experimental colony. An annotation of the red and white regions is proposed from the colony segmentation and its regions corrected in order to satisfy the purity constraint (see Sect. 2.1.2) (color figure online)

Once individual colonies and their segmentations are extracted from the full-size images, the goal is to classify each colony as $[PSI^+]$, $[psi^-]$ or sectored. Figure 3 shows the annotation procedure for counting and quantifying sectors in each detected colony. The procedure here uses the colony segmentation as input, constructs and analyzes a proposed annotation or “idealized" sectoring using the colors of the colony regions, then uses the properties of the annotation to classify the colony.

We make a few assumptions about the colony segmentations in order to classify colonies in our experimental images. Since colonies appear circular, we first assume that the colony segmentations are sufficiently circular and that the center of the colony is also the center of the image. Since the red and white regions of colonies in the experimental images appear sector-like by visual inspection, we also assume that each red and white region of the colony originate from the center and expand outward with linear edges, forming the edges of a geometric sector. Finally, we assume that the colony boundary forms the arc of each sector-like region, which closes and bounds each region.

The following process uses these assumptions to propose idealized regions (see Fig. 3a) for each colony. Given a colony segmentation, we first decompose it into its interior and boundary components. A pixel in the colony segmentation is considered a boundary pixel if it is a colony pixel that is also adjacent to a background pixel. Otherwise, that pixel is considered to be an interior pixel. For simplicity, we skeletonize the boundary of the colony so that it has pixel width 1. Next, we further decompose both the interior and boundary components of the colony respectively into their red and white regions, and then find the connected components of red and white pixels separately on the boundary. For each component, we construct an “idealized" sector (see Fig. 3a) whose boundaries are represented using the component itself as the arc and two lines connecting the endpoints of the arc to the colony center.

To approximate where to draw the lines representing the other two boundaries of an idealized sector, we proceed to find the endpoints on the arc using two methods. This relies on there being no more than 2 endpoints for each skeletonized boundary. We first use the hit-miss algorithm within the SciPy package (Virtanen et al. 2020) to find the endpoints of the skeletonized boundary. For the second case, we scan each pixel on the skeletonized boundary and label a pixel as an endpoint if there is exactly one other boundary pixel adjacent to it. Note that this brute force method is capable of finding endpoints on a corner of a skeleton, while the hit-miss algorithm is capable of finding endpoints near a corner. We then take the union of endpoints located from both methods, because initial observations suggest they correct each other’s shortcomings.

The remaining two boundaries of the idealized sector are then drawn using Bresenham’s line algorithm (Bresenham 1965) to connect the endpoints with the colony center via lines in pixel space, resulting in a closed shape representing the entirety of the idealized sector boundary, while the collection of pixels within represents the interior of the sector. This process is repeated for all red and white regions in the colony, resulting in the full initial annotation representing the regional breakdown of the colony.

#### Purity Metric

Since we assume each region in a colony appears sector-like, we attempt to quantify how well colony segmentations meet this assumption, then perform an additional step for regions which are inconsistent with this assumption. To quantify a region of a colony, we need to analyze the physical structure of the region itself and use simple methods to address inconsistent structure present in the segmentation. To that end, we define a metric we call “purity" to denote the proportion of pixels in each red/white region that are of the same class to measure how well each proposed region and the aggregation of the regions in a colony resemble well-defined sectors.

We first define purity in terms of a single region of a colony. After creating the regions as described in Sect. 2.1, the color of the region (red or white) is assigned to be the same color as the pixels in the region along the boundary of the colony. If we have a sectored colony with *a* red regions and *b* white regions (see Fig. 3a), we denote the red regions as $R_1, \cdots , R_a$ and the white regions as $W_1, \cdots , W_b$. Next, we denote the function *N* to be the number of pixels in a region that have a given color. For instance, we define $N(R_i, red)$ as the number of red pixels in region $R_i$, and $N(R_i, white)$ as the number of white pixels in region $R_i$. Since these are the only two colors for colony pixels in our segmentations, the total number of colony pixels in the region is therefore the sum: $N(R_i, red) + N(R_i, white)$. We then define the purity, *p* of region $R_i$ with respect to the red pixels as

1 $$\begin{aligned} p(R_i, red) = \dfrac{N(R_i, red)}{N(R_i, red) + N(R_i, white)}. \end{aligned}$$

Similarly, we define the purity of region $W_j$ with respect to the white pixels as

2 $$\begin{aligned} p(W_j, white) = \dfrac{N(W_j, white)}{N(W_j, red) + N(W_j, white)}. \end{aligned}$$

Equations (1) and (2) are also described as the proportion of colony pixels within the region that are red or white respectively, and thus give values between 0 and 1, where values closer to 1 indicate the estimated region in the colony is more sector-like with respect to the color of the region, and values far away from 1 indicate the region is far from an idealized sector based on our assumptions.

To define purity for an entire colony, we apply weights to each region to account for size differences between the regions. We first define $N_R$ and $N_W$ to be the number of pixels across all red regions and all white regions respectively, i.e.

3 $$\begin{aligned} \begin{aligned} N_R&= \sum _{i=1}^{a}\left[ N(R_i, red) + N(R_i, white) \right] , \\ N_W&= \sum _{j=1}^{b}\left[ N(W_j, red) + N(W_j, white) \right] . \end{aligned} \end{aligned}$$

Without loss of generality, for each region $R_i$ and $W_j$, we assign weights, $\mu (R_i)$ and $\mu (W_j)$, where

4 $$\begin{aligned} \begin{aligned} \mu (R_i)&= \dfrac{N(R_i, red) + N(R_i, white)}{N_R + N_W}, \\ \mu (W_j)&= \dfrac{N(W_j, red) + N(W_j, white)}{N_R + N_W}. \end{aligned} \end{aligned}$$

We then define colony purity, $p_w$, as the weighted average over all regional purities, i.e.

5 $$\begin{aligned} p_w = \sum _{i=1}^a p(R_i, red) \mu (R_i) + \sum _{j=1}^b p(W_j, white) \mu (W_j) \end{aligned}$$

or equivalently,

6 $$\begin{aligned} p_w = \dfrac{\sum _{i=1}^a N(R_i, red) + \sum _{j=1}^b N(W_j, white)}{N_R + N_W}. \end{aligned}$$

Just like in Eqs. (1) and (2), (6) above takes a value between 0 and 1, where values closer to 1 indicate the estimated regions in the colony are collectively more sector-like with respect to the output segmentation. On the contrary, if $p_w$ is far from 1, this indicates that the estimated regions do not completely capture idealized sectors.

#### Purity Correction

Due to potential pixel-level classification errors in the segmentation step, shapes of each region in the colony segmentation may not sufficiently resemble idealized sectors. Here, we include a procedure to identify inadequate regions by using the value of the purity metric to perform a “correction" of those region with respect to the colony segmentation. This results in a proposed regional annotation capturing standout regions in the colony segmentation (see Fig. 3b).

We assume that the red and white regions have been estimated and the purity for each has been obtained using Eqs. (1) and (2). We then impose a constraint on the purity of each region such that we require at least 50% of a region’s pixels to be of the same color as the region itself to satisfy our assumption that the region is adequately sector-like. If this constraint is not met for a region, then we swap the labels of the pixels on the region’s boundary which in turn changes the color assigned to the region. Mathematically, without loss of generality, if region $R_i$ has a purity of less than 0.5 (i.e. $p(R_i, red)<0.5$), then region $R_i$ has more white pixels than red pixels. For such regions, we change the labels of the pixels along the arc of region $R_i$ corresponding to the colony boundary from red to white. As a consequence, modifying the color of the boundary leads to changing the assigned color of the region from red to white. This process is repeated for all red and white regions independently. Following this procedure, regions are merged if their corresponding boundary pixels are of the same color (see Fig. 3b). By using the mediant inequality as shown in Eq. (7), if the purity of each of these regions is at least 0.5, then the resulting merged region will also have purity greater than 0.5. For example, if there are *n* red regions adjacent to each other following the correction, then

7 $$\begin{aligned} 0.5\le &  \min _{1 \le i \le n} p(R_i, red) = \min _{1 \le i \le n} \dfrac{N(R_i, red)}{N(R_i, red) + N(R_i, white)}\nonumber \\\le &  \dfrac{\sum _{i=1}^n N(R_i, red)}{\sum _{i=1}^n \left[ N(R_i, red) + N(R_i, white)\right] } \le \max _{1 \le i \le n} \dfrac{N(R_i, red)}{N(R_i, red) + N(R_i, white)}\nonumber \\\le &  \max _{1 \le i \le n} p(R_i, red). \end{aligned}$$

If there were any changes made to regions that did not satisfy our constraint, we then repeat the procedure as described in Sect. 2.1 to propose a regional annotation of the colony accounting for the swapped boundary pixels, and recompute the purity for all regions in the colony segmentation. This procedure is repeated until we obtain a proposed regional annotation of the colony where all regions satisfy our constraint.

At the conclusion of purity correction, the color of the pixels on the outer boundary for each independent region will be the same color as the majority of pixels in those regions. We then use Eq. (6) as described in Sect.  2.1.1 to score how well the proposed regional annotation collectively captures sectoring behavior in the colony.

#### Class Assignment

Upon obtaining annotations of colonies whose regions all meet the condition described in Sect. 2.1.2, the number of red and white regions remaining are used to assign a qualitative class on each colony. Colonies with no red regions and at least 1 white region are labeled as $[PSI^+]$. Colonies with at least 1 red region but have no white regions are labeled as $[psi^-]$. Colonies that have at least 1 red and white region are labeled as sectored. In addition, sectored colonies are given a secondary label indicating frequency of sectors. A sectored colony is labeled as S1 if it has one sector, S2 if it has two sectors, and so on.

### Training (Image Segmentation)

Due to the lack of hand annotated colony images, we turn to training a neural network with synthetic images where it is possible to efficiently create ground-truth masks labeling each pixel. An example of a synthetic image generated with its corresponding ground-truth mask is shown in Fig. 8. The objective of this approach is to generate sets of synthetic images of yeast colonies which exhibit key features of the yeast colonies found in the experimental images.

The key features in the images we consider for this work apply to the colonies and the background information. For the colonies, these features consist of circular colony shapes where each colony exhibits sectored red and white regions with slight color variations. We use two representative colors (1 red and 1 white) to fill each circle representing the colony, where the circle is filled with the white color and the red sectors are overlayed. For the background, these features include the colors of the plate, the table on which the plate rests and the border of the plate where aberrations are present, each of which exhibit slight color variations. We choose a representative color independently for each of these three features.

Two corresponding ground-truth masks are generated alongside each synthetic image representing a pixel-by-pixel segmentation of the synthetic image and frequency of sectors per colony respectively. The first mask is created by thresholding the synthetic image, with each pixel in the mask depicting the true label of every pixel (red, white, background). The second mask is generated by placing a small non-zero region at the center of colony, whose intensity is greater when more sectors are present. For simplicity, all the synthetic training images used here have exactly one sector in each colony (see Sect. 4 for more information). More details pertaining to the process for generating the synthetic images with corresponding ground-truth masks is described in Appendix A.2.

After the masks are created, the synthetic image is subject to Poisson noise to introduce slight color variations that are observed in the experimental images (see Sect. A.1). Both the synthetic images and the masks are each saved with size 1024$\times$1024. We then use our synthetic images to train a modified U-Net. (For details see Appendix A.4) A total of 200 synthetic images with their two corresponding masks were generated for this study using the process described in this Section. Out of these images, 150 were used directly for training U-Net, while the remaining 50 were set aside for validation. We use Google Colaboratory to train our U-Net architecture on the 150 images. (Additional details on model training are given in Appendix A.4) After training U-Net, we use the experimental images as input to obtain an output segmentation for the classification step of the [*PSI*]-CIC algorithm. Since our experimental images do not include pixel-by-pixel ground-truth annotations, the quality of the segmentations were judged by eye before a usable set of parameters for U-Net was used for the final version of our [*PSI*]-CIC algorithm.

### Evaluation

Labels are assigned to each colony at the end of the [*PSI*]-CIC algorithm (Sect. 2.1)) such that the conditions described in Sect. 2.1.2 were met. [*PSI*]-CIC predicts colonies with only one colored region as either $[PSI^+]$ if they were purely white or $[psi^-]$ if they were purely red. Colonies that have at least one red and one white region are first labeled as sectored, then are assigned a secondary label indicating the frequency of sectors. We note that sector number is not the only feature of interest, but we use it as a simple way to assess performance (see Sect. 4). In the experimental images, sectored colonies have at most five sectors, so the possible classes assigned to sectored colonies are S1, S2, S3, S4, and S5, denoting both a sectored colony with its frequency of sectors. We evaluate the performance of [*PSI*]-CIC by comparing the proportion of extracted quantifiable colonies whose true labels match those predicted by [*PSI*]-CIC, both with and without the secondary label for sectored colonies.

## Results

Here we present results on the performance of [*PSI*]-CIC on segmenting and classifying colonies from the images used in this work. Section 3.1 provides results on the segmentation and classification of colonies found within the training images. Section 3.2 presents results on the segmentation and extraction of quantifiable colonies, indicating how much of the annotated colony data [*PSI*]-CIC was able to isolate. Section 3.2 provides results on the classification performance of [*PSI*]-CIC. We show that our method is sufficiently accurate at classifying colonies as $[PSI^+]$, $[psi^-]$ or sectored.

### Training Images

**Fig. 4:**
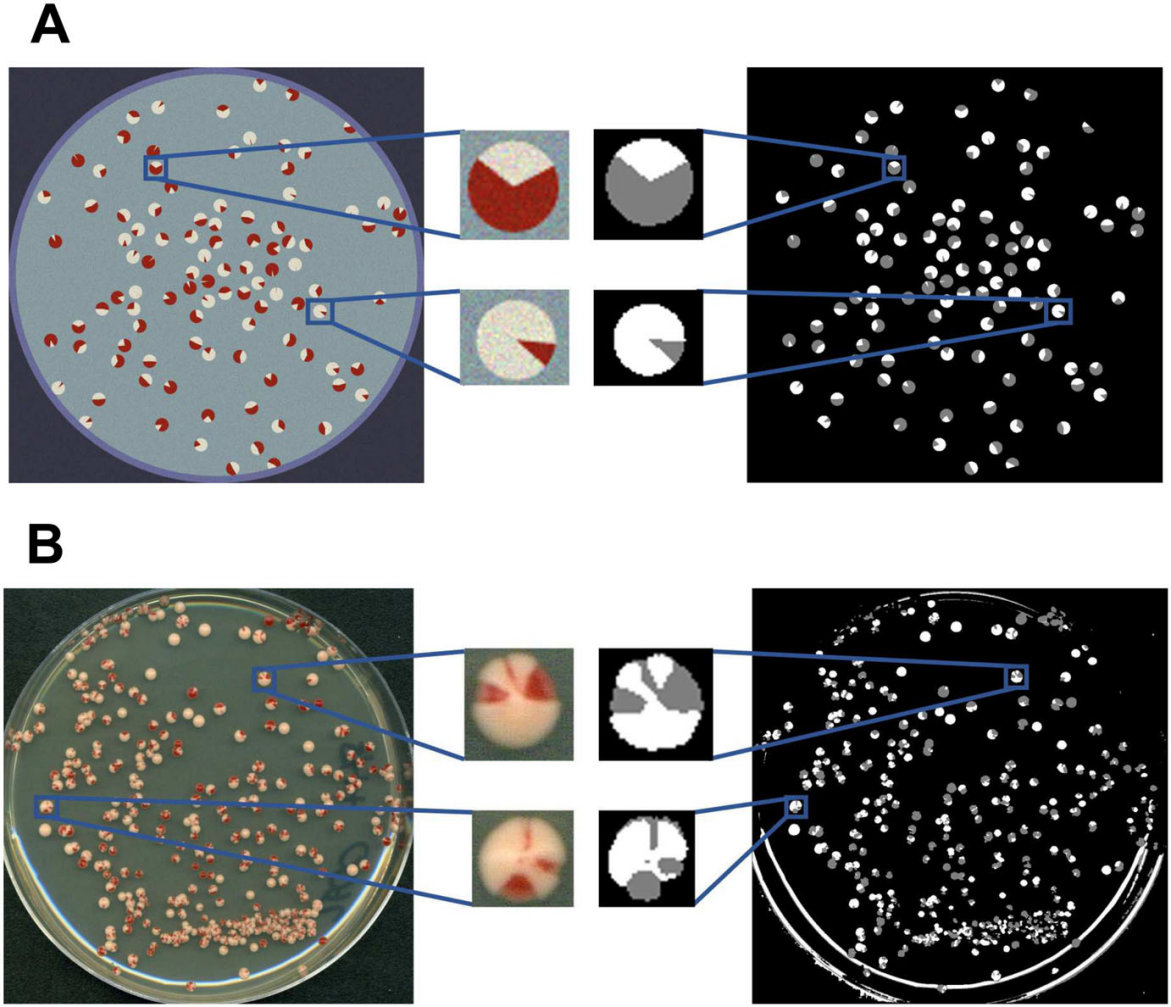
Plate level segmentations. **a** Example of a synthetic image (top left) and its corresponding output segmentation (top right) from the trained U-Net, with two isolated colonies shown up close. The U-Net segmentations have the following color code: Background pixels are black, red colony pixels are gray, and white colony pixels are white. **b** Output for U-Net using one of the experimental images as input. In the middle are the original representations and corresponding output segmentations from U-Net for two colonies from the image (color figure online)

Figure 4 A shows an example of one synthetic image and its corresponding segmentation with distinguishable colonies. From the 150 images used to train U-Net, we obtained a cross-entropy loss of 0.0022 and achieved a segmentation accuracy of 99.96% for the training and validation images after 24 epochs. Approximately 12,786 colonies from the synthetic images were extracted for classification. The remaining 7,214 colonies were excluded since their centers were predicted to be within 150 pixels from the border of the image.

When only the number of connected components of red and white boundary regions were considered, approximately 98.2% of those colonies (12,250 colonies) were correctly classified as having exactly one sector, while the other 236 colonies were incorrectly classified as either $[PSI^+]$ or $[psi^-]$. When our purity correction scheme is applied, the prediction accuracy drops to 95.8%, with 547 colonies incorrectly classified as either $[PSI^+]$ or $[psi^-]$. Upon closer inspection of the incorrectly classified colonies, we found that colonies predicted as $[PSI^+]$ had no more than 4% of their composition as red and colonies predicted as $[psi^-]$ had no more than 4% of their composition as white, regardless of whether purity correction was applied. This suggests that the classification accuracy of our proposed model requires a size threshold on each sector in order to be detectable.

**Table 1:** Isolating quantifiable colonies

Plate	True	Detections	TP	FN	FP	Precision	Recall
1	355	322	243	112	79	0.755	0.685
2	190	156	122	68	34	0.782	0.642
3	269	318	186	83	32	0.853	0.691
4	236	283	192	44	91	0.678	0.814
5	177	187	151	26	36	0.807	0.853
6	127	139	121	6	17	0.877	0.953
7	106	122	106	0	16	0.869	1
8	92	98	81	11	17	0.827	0.88
9	127	101	91	36	10	0.901	0.717
10	112	119	105	7	14	0.882	0.938
11	131	136	120	11	16	0.75	0.779

Breakdown of the number of colonies found on each plate from the images used in this study. For each image, “True” is the total number of colonies in the image that a biologist performing manual counting would be considered quantifiable, or colonies that could be analyzed with simplicity. “Detections” is the number of objects considered for classification, regardless of whether or not they were of quantifiable colonies. Following image segmentation, “TP” (True Positives) is the number of quantifiable colonies extracted, “FN” (False Negatives) is the number of quantifiable colonies that were not detected, and “FP” (False Positives) is the number of non-quantifiable colonies detected. Precision is defined as TP/(TP$+$FP), which is the proportion of all detections consisting of quantifiable colonies. Recall is defined as TP/(TP$+$FN), which is the proportion of all quantifiable colonies detected.

### Testing Images

Before applying the full pipeline to our real plates, we examined if our pre-trained U-Net was sufficient at pixel level segmentation on our real images. A possibility we considered was performing additional processing on the raw images. Because we have no ground truth data sets, this comparison was done by visual examination by computational and biological researchers. For image set 1 (see Fig. 7left) we observed the image resolution was sufficient that the pixel level segmentation appeared to distinguish colonies from background, although there were edge effects. However, for image set 2 (see Fig. 7right) we found pixel level segmentation was improved after we employed a color transfer method as described in Appendix A.1.

Following the execution of the circle Hough transform on these images, we detected approximately 1,981 circular objects (1266 in image set 1 and 715 in image set 2) (see Table 1). We note that almost all of the colonies near the edge of each plate were ignored as they were difficult to discern structurally. From these objects, 1,585 were inspected to be of quantifiable colonies. Approximately 38 circular objects (which included 30 quantifiable colonies) had ill-defined estimated regions and thus were excluded from further analysis. After this, we had 1,555 quantifiable colonies which we classified and compared against manual colony annotations.

**Fig. 5:**
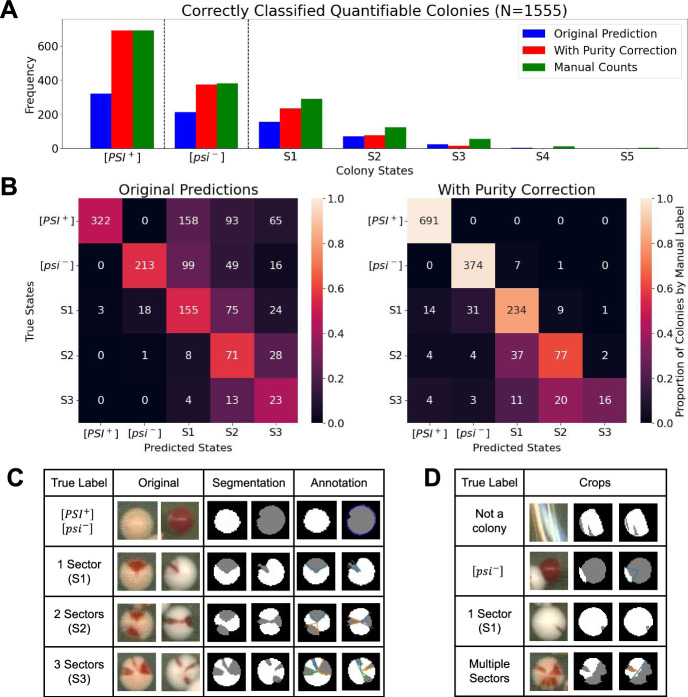
Accuracy of colony-level predictions on quantifiable colony data. **a** Total number of colonies of each class correctly classified. Blue and red bars indicate the number of colonies correctly classified without and with purity correction respectively. The height of the bars represent the number of colonies correctly classified, with the maximum number of colonies possible for each class indicated by the green bars. **b** Confusion matrices showing the frequency of correct and incorrect predictions with our pipeline without (left) and with (right) purity correction applied. The color of each cell indicates the percentage of colonies with the same ground-truth class that were assigned a predicted class through our pipeline. Since [*PSI*]-CIC did not predict colonies to have more than 3 sectors, the 15 colonies whose true labels are S4 and S5 are not included here. **c** Some examples of colony segmentations and annotations for $[PSI^+]$, $[psi^-]$ and sectored colonies along with the frequency of sectors (S1, S2, S3). **d** Some examples of segmentations and annotations for detected objects whose predictions were incorrect or which have no ground-truth class (either not quantifiable or not a colony) (color figure online)

From the quantifiable colonies, 415 colonies were predicted to be sectored, with the number of sectors predicted ranging from 1 to 3. Approximately 89.5% of the quantifiable colonies across all image sets used in this work were classified the same as those manually annotated (Fig. 5a). For colonies labeled as homogeneous, 691 were labeled as $[PSI^+]$ and 374 as $[psi^-]$ (Fig. 5b). In contrast, if we only count the number of connected components on the boundary without performing purity correction, we obtain only a 50.4% accuracy in predicting colony states, demonstrating that our purity correction scheme in [*PSI*]-CIC performs better for estimating regions in the colony segmentations.

**Table 2:** Classification performance

Class	Precision	Recall	$F_1$ Score
$[PSI^+]$	0.969	1	0.984
$[psi^-]$	0.908	0.979	0.942
Sectored	0.981	0.876	0.925
S1	0.788	0.810	0.799
S2	0.694	0.621	0.655
S3+	0.5	0.5	0.5

Table of Precision, recall, and F1 score for each class on quantifiable colonies predicted with [*PSI*]-CIC. For each class, the following definitions apply independently: True positives (TP) are colonies whose predicted class and ground-truth class match. False positives (FP) are the set of colonies with one predicted class, but whose manually annotated class is different. False negatives (FN) are colonies not predicted to be of a given class, but were manually annotated with that class. Precision is defined is TP/(TP+FP), representing the number of colonies correctly predicted to be of the given class, divided by the number of colonies assigned this class. Recall is TP/(TP+FN), representing the number of colonies correctly predicted to be of the given class, divided by the number of colonies manually annotated with the given class. The $F_1$ score is the harmonic mean of both precision and recall. The bottom three rows present the same measures but additionally include frequency of sectors predicted for colonies as a condition for being counted as TP

We use confusion matrices to see how both sector counting schemes place colonies into the correct groups in more detail across both image sets (Fig. 5b). We clearly see that including our purity correction scheme places more colonies on the main diagonal of the matrix. Surprisingly, all quantifiable colonies detected which were manually annotated as $[PSI^+]$ were correctly predicted to be $[PSI^+]$. This was not the case when purity correction was excluded. All but nine of the colonies manually annotated as $[psi^-]$ were correctly classified, with the incorrectly classified ones labeled as S1 or S2. When purity correction is not applied, this method significantly overestimates the number of sectored colonies whose manually annotated class is $[psi^-]$. For sectored colonies, our purity correction scheme shows improved accuracy in classifying colonies with one or two sectors, but slightly less accuracy in classifying colonies with three or more sectors. Fifteen colonies from those extracted were manually annotated as 4-sectored (S4) or 5-sectored (S5), but were not predicted with these classes.

Sectored colonies whose predicted class differs between those predicted without and with purity correction have their predicted frequency of sectors reduced as part of the correction scheme. This suggests our purity correction scheme is sufficiently preventing overcounting of the number of regions per colony in our dataset. Figure 5 C shows the segmentations and regional annotations of a few colonies which were correctly classified. Examples of colonies which were either non-quantifiable or incorrectly classified are shown in Fig. 5d.

The accuracy of colony classifications within each class is shown in Table 2. From the images we used in this study, we found that all quantifiable colonies extracted which were manually annotated as $[PSI^+]$ were correctly predicted as $[PSI^+]$, hence recall for this class was 1. The source of precision being less than 1 is due to a small number of 1-sector colonies being classified as $[PSI^+]$. Similarly, recall for $[psi^-]$ colonies was close to 1 due to some being incorrectly classified as sectored, and precision being less than 1 due to a subset of manually annotated sectored colonies being incorrectly classified as $[psi^-]$. Interestingly, while the accuracy in correctly predicting sectored quantifiable colonies is not as impressive, this category has the highest precision, indicating that the highest proportion of colonies predicted to be sectored were also manually annotated as sectored. However, when considering the frequency of sectors in these colonies, performance degrades with higher frequency of sectors as shown in the bottom half of Table 2.

**Fig. 6:**
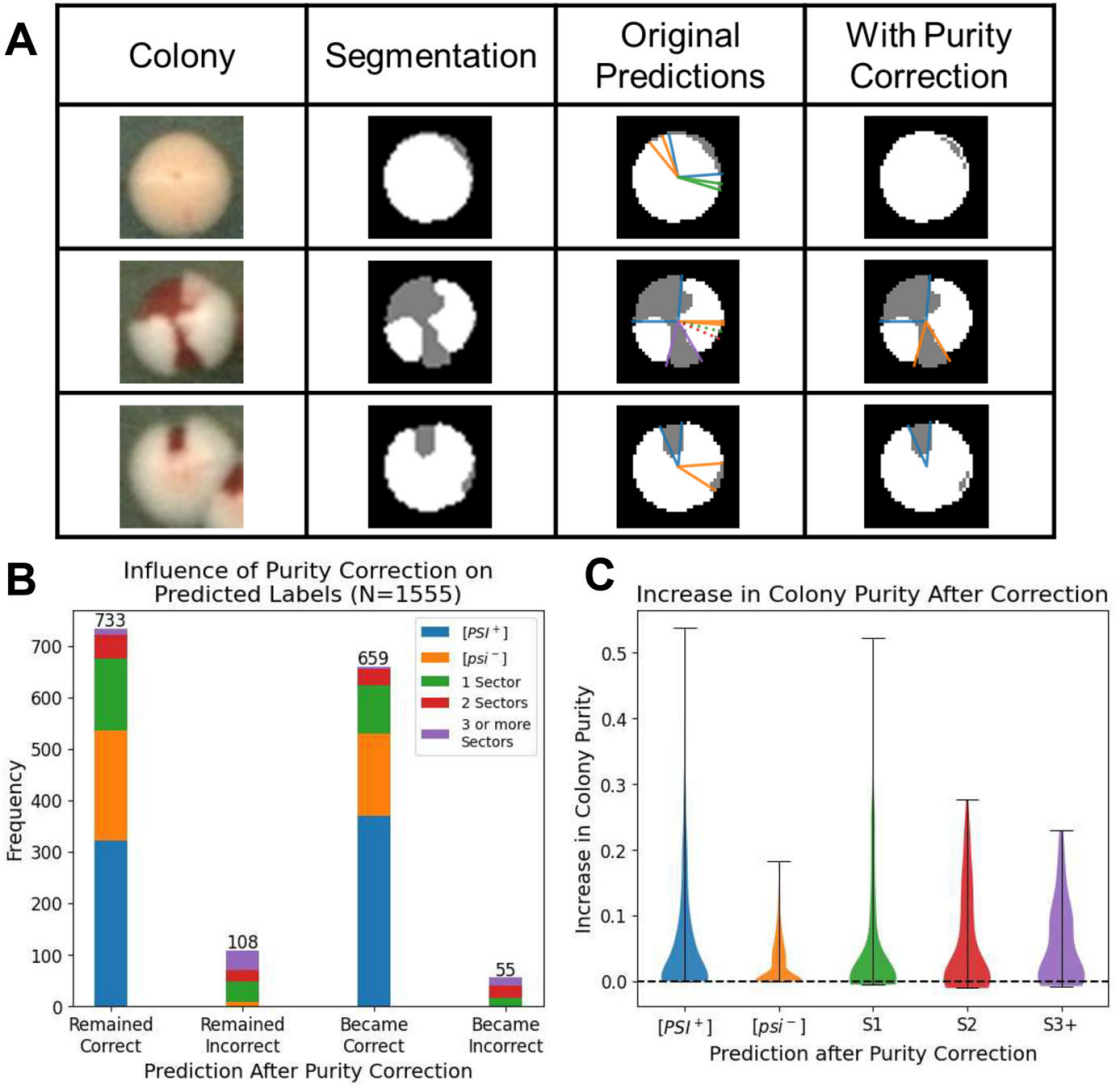
Purity correction improves classification. **a** Example colony segmentations with annotated regions before and after purity correction. **b** The predictions on the quantifiable colonies before and after purity correction, partitioned by their manually labeled states. “Remained Correct” is the set of colonies whose classifications both before and after purity correction matched their manually annotated classes. “Became Correct” is the set of colonies which were incorrectly classified before purity correction, but were correctly classified following purity correction. **c** Violin plots representing the distributions of differences between purities of colonies with and without purity correction, with positive differences indicating higher purity when correction is applied. Horizontal bars indicate the minimum and maximum differences for each subset of classified colonies based on their predicted state and sector frequencies (color figure online)

**Fig. 7:**
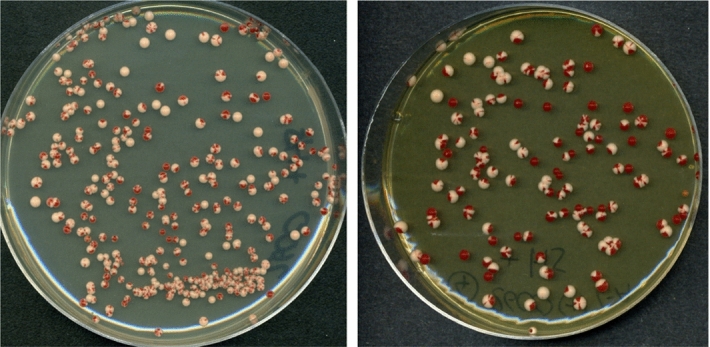
Yeast colony images. Example images of yeast colony plate 2 from image set 1 (left) and plate 8 from image set 2 (right) (color figure online)

**Fig. 8:**
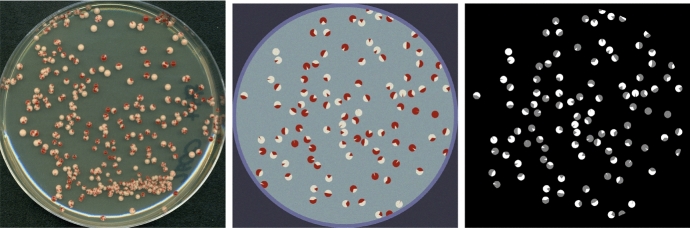
Synthetic Representation of Experimental Images. (Left): Experimental image of yeast colonies with both red and white phenotype obtained with permission from the Serio lab. (Middle): Synthetic image of yeast colonies with both red and white phenotypes generated using Matlab. (Right): The ground-truth mask indicating the label of each pixel in the synthetic image. Background pixels are black, red colony pixels are gray, and white colony pixels are white (color figure online)

Examples of regional colony annotations before and after purity correction are shown in Fig. 6a. Many colony segmentations which had relatively small red or white regions did not meet the threshold for the purity metric and were thus not counted as separate regions. While the use of our purity correction scheme does alter the classifications of approximately half of colonies classified, our results show approximately 42% of all previously misclassified colonies became correct when purity correction was applied (Fig. 6b). In contrast, a subset of 55 colonies were classified incorrectly with purity correction when the original predictions were previously correct, yet the performance of our pipeline outweighs this disadvantage. In nearly all the colonies classified, the proposed regional annotations of the purity corrected colonies exhibit a higher weighted purity (Fig. 6c), as this was one of the objectives of our purity correction scheme as described in Sect. 2.1.2. Based on this information, our method is able to better capture sector-like regions in the colony segmentations which in turn improves accuracy in colony classification.

## Discussion

Two aims of our pipeline for localizing colonies are to find all manually annotated colonies, and to suggest a way to classify colonies where manual annotations are not reliable. One objective [*PSI*]-CIC achieves is ensuring high recall, allowing for extraction of as many quantifiable colonies as possible from manually annotated data, thus satisfying the first aim. While we note that the precision for detecting quantifiable colonies is not very high (see Table 1), such performance is expected because nearly all colonies in the images have a sufficient degree of circularity, not just quantifiable ones. As a result, our method extracted and provided reasonable predictions for approximately 400 additional colonies from the images which were not considered quantifiable. As such, [*PSI*]-CIC could be used as an additional aid in quantifying colonies that are not considered quantifiable to experimentalists. Furthermore, we emphasize that while we use labels S1–S5 for sectored colonies as a way of showing our results, our primary goal with [*PSI*]-CIC is to provide a detailed annotation of individual colonies for biologists to study. The use of labels S1–S5 is primarily for ease of comparison between colonies in our image set while colony annotations provide spatial information on sectors in addition to their frequency. While classification models are suited for predicting the class of an object of interest, it is challenging to also obtain spatial information on the object, motivating our choice to design our algorithm to separately address the problem of locating sectors before classifying colonies.

We observed a few major factors present in the colony images which had an influence on classification accuracy. First, we noticed that many colonies had at least one red or one white region comprising less than 5% of the colony area. As a result, our purity correction method in [*PSI*]-CIC did not accurately isolate these small regions. We believe this is likely a consequence of low image resolution. Near the center of the colony, it is possible for multiple sectors to occupy the same pixel, making it appear in the output segmentation that a sector may not originate at the colony center. Smaller regions in the segmentation may also not satisfy the threshold of the purity metric as defined in Sect. 2.1.2 and as such our method suggests such regions to be part of an adjacent region.

Second, there were also a subset of non-isolated quantifiable colonies whose individual colonies were classified. Due to our assumption that colonies are circular, adjacent clustered colonies may have overlapping regions present in each colony segmentation. Furthermore, clustered colonies were more likely to be excluded from classification since the circle detection step may have had insufficient information in these regions to detect circles there. Visual inspection also suggests that $[psi^-]$ regions in these colonies have a lower growth rate than $[PSI^+]$ regions, reducing circularity of the colony as a whole. However, this difference did not appear to have a significant effect on the number of colonies detected.

Third, individual colony sizes-or similarly, image sizes-may affect both segmentation and classification accuracy. Previous deep learning based segmentation tasks involving microbial colonies on plates used images with spatial dimensions in the couple thousands for each individual plate to adequately capture colonies within the full range of sizes present (Carl et al. 2020; Ferrari et al. 2017). Capturing colony-level information from images of full plates would typically necessitate having high resolution images in order to ensure the individual colonies have a sufficient amount of resolution needed to exhibit clear sectors. However, the purity correction scheme in [*PSI*]-CIC suggests that this size limitation need not be as strict if the objective is to partially capture estimated sectored regions rather than to fully segment them. Furthermore, *a priori* knowledge of colony sizes relative to the plate should still be used to impose a minimum size limitation for colony images to ensure a sufficient amount of detail is captured in the output of the model.

In contrast with the model proposed by Carl et al. (2020), [*PSI*]-CIC relies on the use of synthetic images for training U-Net to segment real colonies rather than using real images directly. This is a convenient and reasonable strategy for simplifying ground-truth mask generation because colonies in our images appear circular and exhibit mostly geometric sector shapes by visual inspection. However, such a strategy makes it more difficult for semantic segmentation models to generalize to more complex image data. Despite this simplification, [*PSI*]-CIC was still able to sufficiently locate, partition, and classify colonies in the experimental dataset used in our study. One limitation of this present study is that [*PSI*]-CIC has been highly tuned to the image resolutions in our colony image sets and does not use images of differing resolution. Further accuracy for classifying colonies using [*PSI*]-CIC may be possible with images large enough to accurately annotate the interfaces between the colony boundary and the interfaces between its red and white regions, but colonies should be large enough for detection. Since the images used by Carl et al. (2020) are more than 3000 pixels in both height and width dimensions-nearly three times higher than the images used in our study-and colony sizes much smaller in proportion to the plate sizes, such images may be too small to reliably segment and annotate if the plate images are resized to 1024$\times$1024. As such, a direct comparison of classification results between [*PSI*]-CIC and that of Carl et al. (2020) using their dataset and ours will not be feasible unless both models are capable of sufficiently classifying images of the same dimensions. We believe it is possible to tune [*PSI*]-CIC to analyze images of higher resolution to make a direct comparison, as the creation of synthetic images of different sizes is simple to implement in our approach. In addition, while our algorithm may potentially utilize higher resolution images to obtain scalably higher resolution colony annotations, computational scalability is a limiting factor requiring suitable hardware. Future work should address consistency of results with respect to image size and resolution to ensure a direct performance comparison could be made between [*PSI*]-CIC and Carl et al. (2020) as well as other similar image classification models which could be adapted.

The use of traditional feature-based approaches to address our specific problem were insufficient for our image data. One direction we have tried was converting each image to a single-channel scale (grayscale) to simplify colony detection, but we found that many $[psi^-]$ and sectored colonies were not being recognized. Two hypotheses are that these colonies blend in with the background when images are converted to grayscale and significant variation in colony phenotype affects the performance of feature-based methods. Furthermore, $[PSI^+]$ is a whole spectrum of colony colors between red and white. We believe tuning a deep learning model such as U-Net on images encompassing this spectrum of variation will allow for more streamlined analysis compared to feature-based methods that do not account for the amount of heterogeneity present in our image data. We note that the sector annotation pipeline we use is highly constrained to the task of locating red and white regions in each colony. Alternate approaches such as clustering of pixels based on color, may be more appropriate for features of interest which exhibit a spectrum of colors (Weller et al. 2024). We emphasize that the goal of our approach was not to solve all colony annotation problems, but to advance the ability to quantitatively assess sectored [*PSI*] colonies.

The use of synthetic images for training CNNs is useful for improving image segmentation and classification when the quantity of annotated data is insufficient and the synthetic images capture sufficient variation present in the desired images to be segmented. While our synthetic images primarily capture the geometric features present in the experimental images, these features vary quantitatively across the experimental dataset. We point out three sources of variation which could be addressed to boost complexity of the synthetic images. First, while the synthetic images account for most of the color variation present in image set 1 as described in Sect. A.1, they do not account for the color variation in image set 2 because the images in this set needed to be pre-processed before they were passed to U-Net for segmentation. An ideal sample of synthetic images should have similar color distributions as in the experimental images. Otherwise, U-Net would need to be independently retrained for each distinguishable set of images. The second source of variation consists of different colony sizes among the synthetic and experimental images. The colonies in our synthetic images have equal sizes, whereas the experimental images have a range of sizes. The third source of variation is the frequency of sectors present in each colony. While each of the synthetic images all contain colonies with exactly one sector each, sector sizes were allowed to vary between colonies.

Even though our synthetic images do not fully capture all these sources of variation, our results emphasize that our synthetic images contained enough information to train U-Net to locate and segment colony features in the experimental images. In particular, following the U-Net training procedure using the synthetic images as described in Sect. 2.2, our results (Sect. 3.2) show [*PSI*]-CIC is adequately capturing sector-like regions in experimental colonies and further classifying them as described in Sect. 2.1. We further note that while color and size are the two primary sources of variation in our images, other sources are possible. For instance, in our approach to training U-Net, our synthetic images contained colonies with at most one sector. We chose this because visual inspection of the data used in this work shows that a majority of sectored colonies identified were single-sectored. An extension of our approach would be to train on synthetic colonies with frequencies of sectors matching the real data available. In addition, while we acknowledge that using colony data capturing more complex colony and sector morphologies will be useful for training our algorithm, the analysis of our algorithm on this type of data lies outside the scope of this present study. In Klaips et al. (2014), sectoring patterns change under distinct treatment conditions, so it will be useful to tailor a training set that incorporates prior available knowledge on the outcomes of different treatments on yeast colonies. More structured and more diverse training data is needed to incorporate additional sources of variation present with experimental images and to ensure robust performance of [*PSI*]-CIC across multiple experimental conditions.

We emphasize that our [*PSI*]-CIC algorithm is designed to provide a solution for quantifying prion phenotypes in yeast from colony-level image data. While our algorithm is extensively tuned to identify $[PSI^+]$ and $[psi^-]$ colonies, we recognize that tuning to additional prion strain datasets which exhibit colony-level phenotypes will be a useful extension of the applicability of our algorithm to test its generalizability toward recognizing other strains. Furthermore, while our algorithm has been tested on colonies each containing hundreds of thousands of cells, it has not been tested on datasets containing smaller colonies where individual cells are visible and sectors less defined. In both cases, deep learning models with many trainable parameters which utilize image data must have suitable hardware such as a GPU to store and process such data during the training process, a necessary limitation for our present work. Further tuning of our algorithm on datasets outside the scope of this current work will be necessary to address both the capability of the algorithm to generalize to similar data. Doing so will significantly improve the usefulness of our algorithm in laboratory settings to reduce the need for manual quantification of colony data.

We note that the primary features we considered when creating synthetic images for training U-Net involve circles and known colors from experimental images. As such, any other organism which exhibits these physical properties are prime candidates for automating colony classification. A natural extension of our work would be to adapt [*PSI*]-CIC to classify colonies of *Candida albicans* which exhibit a white to opaque color switch (Lohse and Johnson 2009; Sasse et al. 2013) as well as different colony size phenotypes under the same growth timeline (Morschhäuser 2010). Additional types of sectored image data at the colony level such as gene expression data obtained through flourescent-based assays (Liu et al. 2016; Hallatschek et al. 2007) could be incorporated to develop methods for spatial structural analysis of such data. These considerations warrant a further generalizability study on the usefulness of [*PSI*]-CIC in segmenting images containing other species of yeast or other circular shaped colonies as a future research direction.

## Conclusion

In this study, we constructed a new computational pipeline we call [*PSI*]-CIC designed for high-throughput segmentation and quantification of sectored yeast colonies found in images of experimental plates. We show that synthetic images could be used for training U-Net to segment colonies from experimental images based on their color and simple shape. Results show that we are able to obtain acceptable colony counts from plated colony images, given that the segmentation adequately captures the circularity and regions of the colony. We demonstrate that [*PSI*]-CIC obtains colony states and sector frequencies comparable to manual annotations from experimentalists. This is the first model designed specifically for quantifying sectors in yeast colonies indicative of changes in prion dynamics within individual cells. The work discussed here is a big step forward for providing researchers a computational framework to gain novel insights into the mechanisms driving prion loss in yeast colonies.

## Data Availability

Code for the [*PSI*]-CIC algorithm and for creating synthetic images for the purpose of training U-Net and the testing images analyzed in our results are available on Github (https://github.com/jcollignon/psi-sectored-classification). The weights for U-Net to obtain segmentation results is also accessible via Github LFS due to its large file size. If there are any issues accessing the weights file, the authors can make this file available upon request.

## Appendix A

### Image Acquisition and Pre-processing

Exponentially growing cultures of the yeast *Saccharomyces cerevisiae* strain 74D-694 *MAT*a: *ade1-14*, *trp1-289*, *his3*$\Delta$*-200*, *ura3-52*, *leu2-3*, *112,* $[PSI^+]$ $[PIN^+]$ were subjected to heat shock at $40^{\circ }$C for 30 min by water bath to induce curing. Approximately 500 cells were then plated onto YPD-Cox media (0.25% yeast extract, 1% bactopeptone, 2% agar, 4% glucose) and grown for 3 days at $30^{\circ }$C followed by 5 days at room temperature to allow colony pigmentation to develop. Images of plates were acquired using an Epson V370 scanner.

A total of 11 images of different plates were acquired and used to test [*PSI*]-CIC. Image set 1 (plates 1–5) contains five images with one plate per image each containing up to approximately 500 colonies (example in Fig. 7left). All the colonies in these images are either white $[PSI^+]$, red $[psi^-]$, or sectored phenotype (a mix of both $[PSI^+]$ and $[psi^-]$). One of these five plates contains a large number of colonies with sectored phenotypes. Image set 2 (plates 6-11) contains six images which are similar to those in image set 1 (example in Fig. 7right), but these images are less saturated overall and four of these plates contain a significant number of sectored colonies present. These images were pre-processed using a color style transfer method (Chia 2019) before further use.

Colonies in each image were hand annotated and sectoring of each quantified by a yeast biologist. Colonies appearing entirely white or red were scored $[PSI^+]$ or $[psi^-]$ respectively. If a mix of red and white pigment was present in a colony it was scored as sectored. Colonies too small to reasonably determine the presence or absence of sectoring were deemed unquantifiable and not considered in our results. If the boundaries of multiple colonies intersect each other in a cluster extensively enough such that half the colony volume is shared, the entire cluster is deemed unquantifiable.

Both image sets 1 and 2 were used for different experiments at different times. One important feature to note across image sets 1 and 2 is variation of color and lighting conditions. Since U-Net is trained on synthetic images whose color is based off the images in set 1, U-Net may not accurately segment colonies from image set 2 because by eye the color profiles are different from what U-Net was trained with. Instead of retraining U-Net to address this issue, we opt toward pre-processing the real images until they appear close to a “standardized" image. We use an implementation of a color profile transfer scheme written by Chia (2019) and adapt it for execution on Google Colaboratory. This code is an implementation of the work by Reinhard et al. (2001) which transforms a source image by applying onto it the color characteristics of a desired “target" image. The objective for this pre-processing step is to ensure that the images in set 2 have similar color features as the images in set 1 so that U-Net will produce similar quality output segmentations.

For the purpose of this work, we chose the target image to be the image of plate 2 in image set 1 (also shown in Fig. 7left). All six images in set 2 (plates 6-11) were used as source images for the color transfer scheme before input to U-Net. No pre-processing was done on image set 1 because these images have the color profiles that U-Net was originally trained on. No adjustments in brightness and contrast were applied to these images before or after the color transfer scheme was applied. While there are subtle differences between the color profiles in the original and pre-processed images in set 2, their output segmentations are significantly different. In particular, the segmentation of the preprocessed images display obvious quality improvements such that many more colonies could be sufficiently discerned for classification. Most of the colonies present in the output segmentations were sufficient enough for the classification scheme as described in Sect. 2.1.

### Synthetic Image Generation

Due to the lack of hand annotated colony images, we turn to training a neural network with synthetic images where it is possible to efficiently create ground-truth masks labeling each pixel. An example of a synthetic image generated with its corresponding ground-truth mask is shown in Fig. 8. This approach involves generating sets of synthetic images of yeast colonies which exhibit key features of the yeast colonies found in the experimental images, which comprise of colonies with sectored red and white regions where the color of each slightly vary. We use two representative colors for the colonies-1 red and 1 white color-to fill each circle representing the colony and the overlying sector. Similarly, we use three representative colors for the background corresponding to the interior of the plate, the border of the plate, and the table on which the plate rests respectively and fill each of these regions with those colors. Each color selected corresponds to an RGB vector [*R*, *G*, *B*] such that $R,G,B \in [0, 255]$.

For each synthetic image, two representations as well as five masks are generated, each with size 1024$\times$1024. The two representations of each image include one with Poisson noise and one without. The images containing Poisson noise are used for training U-Net in Sect. 2.2, while the images without Poisson noise are to simplify the process for creating the associated ground-truth masks. The five masks created label (1) the colony pixels, (2) the white colony pixels, (3) the red colony pixels, (4) the red and white colony pixels merged, and (5) the number of sectors in each colony. The first three masks are created through a series of grayscale conversions and binary thresholding operations on the image at intermediate steps of the process. The fourth mask is used as the ground-truth mask for training U-Net, while the fifth mask is used to assess the accuracy of [*PSI*]-CIC in quantifying the frequency of sectors in each colony (see Sect. 2.2).

For each synthetic image, the process for creating the noisy/noiseless representations and ground-truth masks is as follows:

1. We first initialize the image by changing the color of the background represented by the RGB vector [54, 54, 68]. This element represents the tabletop at which the plate rests.
2. A circle of radius 30 whose center coincides with the image center is generated above the background and filled with the color represented by the RGB vector [137, 155, 160]. This element represents the body of the plate.
3. 100 points are sampled inside the circle generated in step 2 such that the minimum distance between any two points is at least 2. Then, circles of radius 1 are generated whose centers coincide with the sampled points. Each circle is then filled with the color represented by the RGB vector [221, 217, 199]. These elements represent the colonies on the plate.
4. Two circles of radius 29 and 31, each with the same center as the circle generated in step 2 are generated. The space in between the circles is filled with the color represented by the RGB vector [105, 107, 152]. This element represents the part of the background corresponding to the border of the plate.
5. An image of size 1024$\times$1024 is saved temporarily. Then, binary thresholding is performed on the resulting image following a grayscale transformation. The result is the final ground-truth mask representing colony pixels.
6. For each circle generated in step 3, two points are uniformly selected on the circle, and lines connect those two points independently with the center of the circle. The space in between is filled with the color represented by the RGB vector [148, 36, 23]. This element represents the red region of a colony. For circles where *n* sectors will be generated, 2*n* points are uniformly selected, and the process described here is performed for each pair of points along the length of the circle.
7. Step 4 is repeated to regenerate the border above the colonies.
8. An image of size 1024$\times$1024 is saved. The result is the noiseless representation of the synthetic image.
9. Binary thresholding is performed following a grayscale transformation on the image from step 8. The result is the final ground-truth mask representing white colony pixels only.
10. The white colony mask from step 9 is subtracted from the full colony mask in step 5. The result is the final ground-truth mask representing red colony pixels only.
11. Since the red colony pixel and white colony pixel masks are fully disjoint, we merge the two masks, assigning one label to white colony pixels and a different label to red colony pixels while all background pixels are labeled 0. The result is the final ground-truth mask showing the locations of red and white colony pixels and is used for training U-Net.
12. An additional mask is created at the center of each colony which shows a small square whose label is the number of sectors generated plus 1. The result is saved as a mask of size 1024$\times$1024. This represents the true labels for the frequency of sectors in each colony within a synthetic image and is used to assess the performance of [*PSI*]-CIC (Sect. 2.3).
13. Finally, the noiseless image saved from step 8 is given Poisson noise, then saved with size 1024$\times$1024. The result is the noisy representation of the synthetic images that is used for training U-Net (Sect. 2.2).

### Colony Extraction

Implementation of the steps to locate colonies as described in Appendix A.4 is done using the Python package oct2py (Silvester et al. 2011) to allow Octave to run within the environment. Octave’s function imfindcircles is used to implement the circle Hough transform (Hough et al. 1962) for locating circular objects in the output segmentations. This function requires two additional parameters: a range of radii of circular objects to detect, and a sensitivity to allow for the detection of objects with slight circular imperfections. To explore the variability in colony sizes within the experimental images, we first find all connected components of colony pixels in their output segmentations, then for each connected component, we locate clusters corresponding to isolated colonies and estimate their radii individually. To do this, we place a bounding box around each connected component separately. Here, we make the assumption that a connected component in the segmentation corresponds to an isolated colony if it meets the following conditions:

1. The connected component must have a number of pixels between a minimum and maximum value. In our case, we require all connected components to have between 100 and 2000 pixels. This is a way to filter colonies that are too big or too small.
2. The bounding box of the connected component must have an aspect ratio close to 1. In our case, we required the greater ratio between length and width of the bounding box to be less than 1.2 to account for image compression and imperfections in the circularity of colonies in the output segmentation. This is also a filter for removing most clusters of colonies from consideration, especially those whose colonies appear to be co-linear.
3. The proportion of pixels within the bounding box consisting of either red or white colony pixels must be between a minimum and maximum value. In our case, we required that the proportion of pixels inside the bounding box to be between 0.7 and 0.9 which contains $\pi /4$, the ratio between the area of a circle and smallest enclosing square respectively. This helps remove colonies whose circularity is insignificant or are too close to the border of the plate.

The collection of radii is used to estimate a range of radii to use for detecting circular objects in the entire segmented image. Since imfindcircles strongly recommends that circular objects have a radius of at least 5 pixels, we also set an arbitrary minimum of 7 pixels for the radius of circular objects. If the minimum dimension of any bounding box is less than 7, we temporarily rescale the entire segmentation so that the smallest dimension of any bounding box is 7, before using imfindcircles, using the range of radii for the objects in the scaled image. The sensitivity parameter of imfindcircles is set to 0.9 to allow adequately imperfect circular objects in the output segmentations to be detected. Following the implementation of imfindcircles, the radii, center coordinates, and the coordinates of the bounding boxes are recorded and saved in CSV files. If rescaling was done prior to recording this data, the data is rescaled so that it corresponds to the original sized segmentation. Finally, the region within each bounding box is cropped from the image and saved as a separate image for classification.

### *[PSI]*-CIC Implementation and Training

A total of 200 synthetic images and corresponding ground-truth masks were created in MATLAB for use as training data, where 150 of these images are used directly in training and 50 were set aside for validation. For each image, 100 non-overlapping white circles representing colonies were placed within the region representing the plate, then one red sector was placed above every colony in the image. Specific details about the placement of colonies and sectors and generation of ground-truth masks are described in Appendix A.2. For the purpose of classification, each of the 20,000 colonies across all synthetic images were labeled to have exactly one sector. All images and ground-truth masks were saved as PNG files.

The remainder of [*PSI*]-CIC is implemented in an interactive Python notebook with GPU access using Google Colaboratory. Construction of the U-Net architecture was implemented and compiled using the Keras packages in Tensorflow. The network is trained using the synthetic images of size 1024$\times$1024, with a batch size of 1 due to the size of the images used and the amount of computational memory available. We use Tensorflow’s categorical cross-entropy loss function and Adam optimizer. The number of epochs was not predetermined; instead, training stopped when the validation loss decreased by at most 0.001 over a period of 5 epochs. This is a helpful check to prevent the model from overfitting to the image set. The learning rate is initially set to $10^{-4}$, but as the validation loss decreases and reaches a local minimum, the learning rate decreases by a factor of 10, with the minimum learning rate possible being $10^{-6}$. Segmentation accuracy for each image is computed to be the number of pixels whose labels match their corresponding ground-truth labels divided by the total number of pixels in the image, while accuracy over the entire image set is the mean of the individual accuracies.

After each epoch, a check is performed on the validation images to determine if the segmentation accuracy is higher than in the previous epoch; if the accuracy is higher, the new parameters are saved, which could be used as a checkpoint for future training of U-Net. When U-Net is sufficiently trained to segment red and white colony pixels in the synthetic images, we apply it to produce output segmentations of the colonies for each experimental image whose pixels are assigned one of three labels (red, white, or background). The segmentations of each plate are saved as individual PNG files.

Following segmentation of the images, steps for locating colonies are implemented using oct2py (Silvester et al. 2011) in Python which enables the use of Octave functions. The circle Hough transform is done using the Octave function imfindcircles found within the image package and is used to detect circular objects in the resulting output segmentation consisting of clusters of colony pixels (both red and white). The specific use of imfindcircles for locating colonies is described in detail in Appendix A.3. Objects detected close to the edges of the image are filtered out. Information about the size and location of the extracted objects are saved as CSV files, with one file per image. Proposed regional annotations for each colony extracted are constructed as described in Sects. 2.1.1 and 2.1.2, and qualitative classes are assigned to each colony as described in Sect. 2.1.3.
